# Locality-aware pooling enhances protein language model performance across varied applications

**DOI:** 10.1093/bioinformatics/btaf178

**Published:** 2025-07-15

**Authors:** Minh Hoang, Mona Singh

**Affiliations:** Lewis-Sigler Institute of Integrative Genomics, Princeton University, Princeton, NJ 08540, United States; Lewis-Sigler Institute of Integrative Genomics, Princeton University, Princeton, NJ 08540, United States; Department of Computer Science, Princeton University, Princeton, NJ 08540, United States

## Abstract

**Motivation:**

Protein language models (PLMs) are amongst the most exciting recent advances for characterizing protein sequences, and have enabled a diverse set of applications, including structure determination, functional property prediction, and mutation impact assessment, all from single protein sequences alone. State-of-the-art PLMs leverage transformer architectures originally developed for natural language processing, and are pre-trained on large protein databases to generate contextualized representations of individual amino acids. To harness the power of these PLMs to predict protein-level properties, these per-residue embeddings are typically “pooled” to fixed-size vectors that are further utilized in downstream prediction networks. Common pooling strategies include Cls-Pooling and Avg-Pooling, but neither of these approaches can capture the local substructures and long-range interactions observed in proteins.

**Results:**

We propose the use of attention pooling, which can naturally capture these important features of proteins. To make the expensive attention operator (quadratic in the length of the input protein) feasible in practice, we introduce bag-of-mer pooling, or BoM-Pooling, a locality-aware hierarchical pooling technique that combines windowed average pooling with attention pooling. We empirically demonstrate that both full attention pooling and BoM-Pooling outperform previous pooling strategies on three important, diverse tasks: (i) predicting the activities of two proteins as they are varied; (ii) detecting remote homologs; and (iii) predicting signaling protein interactions with peptides. Overall, our work highlights the advantages of biologically inspired pooling techniques in protein sequence modeling and is a step toward more effective adaptations of language models in biological settings.

**Availability and implementation:**

https://github.com/Singh-Lab/bom-pooling.

## 1 Introduction

Protein language models (PLMs) have recently emerged as a new technique for modeling the biological “grammar” that governs the behaviors of proteins (Alley *et al.* 2019, Bepler and Berger 2019, Heinzinger *et al.* 2019, Rao *et al.* 2019, Bepler and Berger 2021, Elnaggar *et al.* 2021, Rives *et al.* 2021, Lin *et al.* 2023). State-of-the-art PLMs have gained attention for their promising applications in many biological tasks, including protein structure prediction from single sequence (Chowdhury *et al.* 2022, Lin *et al.* 2023, Jing *et al.* 2024), predicting various functional properties of proteins (Rao *et al.* 2019, Schmirler *et al.* 2024), and assessing the impact of mutations on protein fitness (Meier *et al.* 2021, Brandes *et al.* 2023). The power of PLMs is often attributed to their underlying transformer architectures (Vaswani *et al.* 2017), which have been adapted from large language models in natural language processing (NLP). When trained on widely available and vast protein sequence databases, transformer-based PLMs learn to generate high-dimensional contextualized vector representations of individual amino acids (AAs); these embeddings capture functional information both at the level of individual sites as well as due to context from other AAs in the sequence.

**Figure 1.**
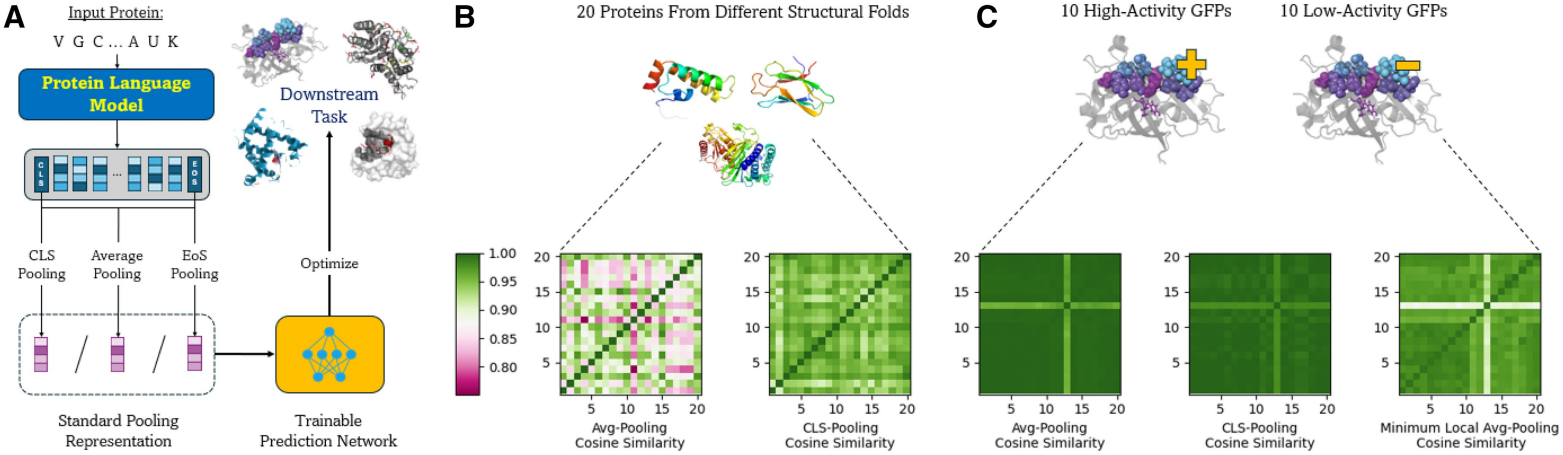
(A) Standard workflow to adapt PLM embeddings for downstream tasks. (B) Heatmaps of pairwise cosine similarities amongst Avg-Pooling and Cls-Pooling representations of 20 proteins sampled from different folds in the SCOPe dataset (Fox *et al.* 2014). Avg-Pooling representations exhibit a range of similarities across across structurally dissimilar proteins, whereas CLS-Pooling does not, suggesting the latter may not be as well suited to differentiate structurally dissimilar proteins. (C) Heatmaps of 20 GFP protein mutants with high and low fluorescence activity levels sampled from the TAPE fluorescence dataset (Sarkisyan *et al.* 2016), comparing Avg-Pooling and Cls-Pooling to the minimum local cosine similarity measure described in Section 3.1. While the latter similarity measure has notably higher similarity amongst the high fluorescence proteins, as is desirable, the other two methods do not. All embeddings were obtained using the ESM-2 model with 150M parameters (Rives *et al.* 2021) and 640 embedding dimensions. Protein illustrations are acquired from SCOPe database (https://scop.berkeley.edu/).

PLMs are often pre-trained independent of any downstream biological task in a self-supervised manner using the masked language modeling objective (Devlin *et al.* 2019). To make PLMs work on a specific application, it is necessary to utilize sufficient labeled task data and either fine-tune these models for this task or train supervised methods using the embeddings produced by the PLMs (Radiya-Dixit and Wang 2020, Elnaggar *et al.* 2021, Jia *et al.* 2022, Schmirler *et al.* 2024, Sledzieski *et al.* 2024). In either case, sequence-level inference tasks typically require practitioners to pool the full PLM embedding sequence into a constant-size representation that fits into a downstream prediction head. For example, Cls-Pooling and Eos-Pooling are reductive pooling techniques that summarize a sequence using the embedding of a single special token added at the beginning or the end of the sequence, respectively. Alternatively, the Avg-Pooling strategy represents a protein sequence by taking the mean of all of its amino acid embeddings (see Fig. 1A).

The above pooling techniques are often applied by default when using PLMs, mainly due to their strong performance in NLP tasks. However, their effectiveness in emerging biological applications of PLMs remains largely unexplored. Indeed, in exploratory analysis, we find significant weaknesses for these pooling techniques, with Cls-Pooling leading to very different proteins having highly similar representations, and Avg-Pooling being less suited to represent protein variants whose functions are driven by a few positions within the sequence (see Section 3.1). Unlike natural language, proteins have specific properties that present unique challenges, including functions that depend on 3D structures; a hierarchical folding organization that leads to long-range inter-dependencies in the linear sequence; and varying significance of local substructures such as domains and motifs.

In this paper, we propose incorporating a learnable attention mechanism (Vaswani *et al.* 2017) within the pooling layer to adaptively focus on relevant regions of the protein sequence or structure and assign higher importance to features or residues that are crucial to understanding its function or interactions with respect to a specific downstream application. Through capturing these local features, the attention pooling layer can engineer features that better reflect protein behaviors, enabling more precise predictions in biological tasks. We provide two variants of this attention pooling layer to accommodate different types of downstream settings, including self-attention pooling for single-sequence supervised learning tasks, and cross-attention pooling for pairwise learning tasks (see Section 3.2). Although attention pooling prioritizes biologically significant patterns, its computational cost is quadratic in the length of input sequences and therefore is less practical than simple techniques such as Avg-Pooling.

**Figure 2.**
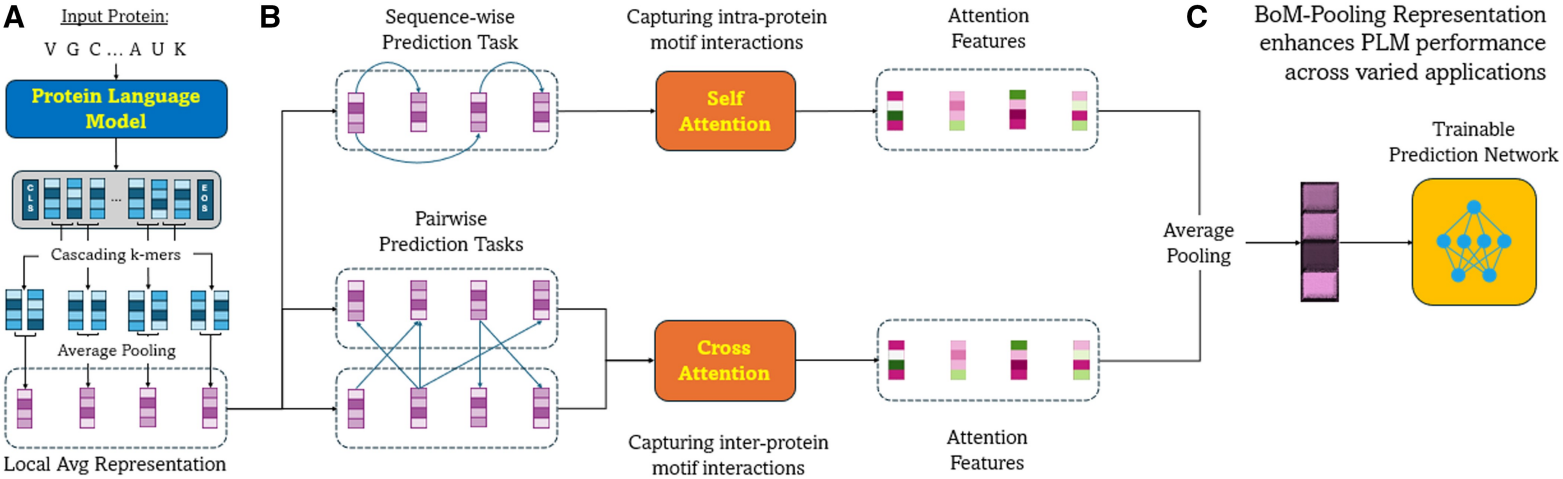
Overview of BoM-Pooling. (A) Input protein is cascaded into overlapping *k*-mers in the PLM embedding space. Avg-Pooling is applied to each *k*-mer to obtain a local AvgPooling representation. (B) Local Avg-Pooling representations are transformed into locality-aware features using learnable self-attention (single-sequence prediction tasks) or cross-attention pooling (pairwise contrastive or interaction detection tasks) (Section 3.3). (C) Attention features are averaged and passed to a downstream network to tackle diverse biological tasks, where BoM-Pooling consistently outperforms other pooling methods such as Avg-, Cls-, and EoS-Pooling (Section 4).

To strike a balance between cost and performance in pooling techniques, we next introduce a novel locality-aware hierarchical pooling technique for PLMs, which we refer to as bag-of-mer pooling (BoM-Pooling, see Section 3.3). Our method first cascades the sequence into smaller overlapping *k*-mers and performs local Avg-Pooling within each window. This set of pooled *k*-mers is subsequently condensed into a fixed-sized vector using the above attention pooling mechanism (see Fig. 2). Supplementary Appendix S1 provides theoretical insights demonstrating that the window pooling approach trades off the compactness of a global Avg-Pooling representation in exchange for greater fidelity to the original PLM embeddings.

We compare the effectiveness of attention pooling and BoM-Pooling to Cls-Pooling, Eos-Pooling, and Avg-Pooling in terms of empirical performance on a wide range of protein related tasks, including predicting fitness of fluorescent proteins (Sarkisyan *et al.* 2016); predicting β-lactamase activity levels (Gray *et al.* 2018); predicting signaling protein-peptide interactions that are mediated by phosphotyrosine binding domains, tyrosine phosphatases and tyrosine kinases (Cunningham *et al.* 2020); and predicting remote homologs (Fox *et al.* 2014) (see Section 4). We consider five PLMs and for each compare the performance of our pooling schemes to existing pooling approaches. In all benchmark tasks, both attention pooling and BoM-Pooling consistently show improved predictive performances over pooling techniques inherited from NLP research. We also show that BoM-Pooling models using long windows and sparse overlap between consecutive windows (i.e. indicating high cost-saving) are able to closely approximate the performance of full attention pooling. That is, the performance of BoM-Pooling approaches that of full attention pooling, while incurring a much lower computational cost. Depending on the protein task and PLM, we find that BoM-Pooling can yield up to a 11.4% median improvement in performance over existing pooling techniques adapted to have the same number of parameters as BoM-Pooling, and an even larger gain over these existing pooling techniques when utilized as they typically are in previous work (see Supplementary Section S4 and Supplementary Appendix S3).

Overall, our main contributions are (i) the introduction of BoM-Pooling, a locality-aware hierarchical pooling scheme based on attention that can capture important local and long range interactions within and across protein sequences; (ii) theoretical insight for considering the trade-off between the compactness of a pooling technique and how well it represents the original PLM embeddings; (iii) empirical evidence across multiple tasks and PLMs that BoM-Pooling yields better predictions; and (iv) a demonstration of how important the choice of pooling method can be. Our work showcases the potential benefits of using a biologically motivated pooling technique in proteomics, and thus paves the way for more successful adaptations of protein language models in the future.

## 2 Preliminaries

### 2.1 Attention mechanism

Transformer-based PLMs, specifically their encoder modules, are constructed via stacking attention layers (Vaswani *et al.* 2017). For a sequence of length *l*, let $H^{\left(0\right)}$, an l×d matrix, denote its initial encoding, where each token is represented in *d* dimensions. We assume that the sequence has been padded with a special classification token [Cls] at the beginning, and an end-of-sentence token [EoS] at the end. The output of the ${\left(i+1\right)}^{\text{th}}$ attention layer with trainable linear transformations Q(·), K(·), and V(·) is given recursively by:


(1) $$H^{\left(i+1\right)}=\text{softmax}\left(\frac{Q\left(H^{\left(i\right)}\right){\left[K\left(H^{\left(i\right)}\right)\right]}^{⊤}}{\sqrt{d}}\right)V\left(H^{\left(i\right)}\right)\;.$$

Let *m* be the total number of attention layers in the PLM encoder. Every hidden representation, up to the final PLM output $H^{\left(m\right)}$, will also have dimension l×d. For ease of notation, we will omit the superscript *m* in subsequent analysis and simply denote the PLM output by $H=[h_{1},h_{2},\ldots h_{l}]$ where $h_{i}$ denotes the $i^{\text{th}}$ token in H. We note that while Q(·), K(·), and V(·) are simple linear transformations in PLMs, mainly for computational reasons, we will consider more expressive architectures for Q(·),K(·),V(·) in the pooling context of this work (see Section 4).

### 2.2 Pooling techniques

As *l* varies with input sequences, a pooling step is required to aggregate these token embeddings into a fixed-size representation before passing it to some downstream predictor network. Padding input sequences to the same length with [PAD] tokens produces a constant-size embedding across all inputs, but it will also require a large predictor network to perform downstream prediction. Popular pooling techniques for PLMs, such as Cls-Pooling, Eos-Pooling, and Avg-Pooling, are global pooling methods that condense H into a single *d*-dimensional vector:


(2) $$P_{\mathrm{CLS}}(H)≜h_{1},\;P_{\mathrm{EoS}}(H)≜h_{l},\;\text{and}\;P_{\mathrm{Avg}}(H)≜\frac{1}{l}\sum_{i=1}^{l}h_{i}\;,$$

which is subsequently passed to a downstream predictor network for supervised training with respect to domain-specific tasks, as shown in Fig. 1A. Very recently, optimal transport has also been used for global pooling (NaderiAlizadeh and Singh 2024). In contrast, techniques such as local Max-Pooling and Local-Avg-Pooling operate on small regions of the input and can yield multiple *d*-dimensional vectors. Max-Pooling and Local-Avg-Pooling have been used in various applications of computer vision (i.e. pooling small patches of pixels in image recognition).

Other trainable mechanisms have been increasingly integrated into pooling strategies to enhance representation learning of local interactions, leading to improved performance in tasks such as machine translation and sentiment analysis (Li *et al.* 2018, Zhang *et al.* 2021). However, these pooling strategies have not been examined in the context of proteins, where prediction tasks may naturally benefit from features that represent local substructures and the interactions amongst them. Independent of pooling techniques for language models, distance preserving embeddings of strings to normed vector spaces (Cormode *et al.* 2000) and minimizer sketches (Hoang *et al.* 2022a, b, 2024) have also been explored as methods for information pooling for sequence data.

### 2.3 Biological benchmark datasets

*Fluorescence intensity prediction (FLUO):* A common goal in protein engineering is to optimize a protein to perform its functions more effectively. In this task, we are given a dataset of 54 025 green fluorescence protein (GFP) variants and their log-fluorescence intensity levels (Sarkisyan *et al.* 2016). The goal is to predict intensity levels, a key step for engineering GFPs with enhanced fluorescence to enable better tracking of proteins in living cells. We use the dataset splits made available in the DeepProtein benchmark (Xie *et al.* 2024) with 21 446 training and 5362 validation sequences, each of which contains fewer than 4 mutations. The test set consists of 27 217 variant sequences with 4 or more mutations.

β *-Lactamase activity level prediction (BLAC):* Predicting the impact of missense mutations on protein function is critical for a variety of tasks, including understanding genotype-phenotype relationships and genetic diseases, and guiding drug design. In this task, we are given a dataset consisting of 5198 variants of the TEM-1 β-lactamase protein and their empirical fitness scores estimating mutation effect (Gray *et al.* 2018). Understanding the functional effects of mutations within this protein is important as the TEM-1 β-lactamase enzyme is responsible for β-lactam antibiotic resistance in gram-negative bacteria (Palzkill and Botstein 1992). We use the dataset splits from DeepProtein with 4158 training sequences, 520 validation sequences, and 520 test sequences.

*Remote homology detection (RH):* One of the most important problems in computational biology is to detect homologs; this is a difficult task when sequence similarity is low. To assess the effectiveness of BoM-Pooling in this domain, we further conduct experiments with the SCOPe dataset (Fox *et al.* 2014), a hierarchical classification of protein structures. We extracted approximately 20 000 proteins between 150 and 1022 AAs in length from the α, β, and α/β structural classes in the SCOPe 2.08 database. Each protein is hierarchically classified into fold, superfamily, and family based on sequence and structural similarities. Proteins within the same family tend to exhibit greater sequence and functional similarity compared to those that are only in the same superfamily or fold. Two proteins are considered homologs if they belong to the same superfamily, and are further classified as remote homologs if they also belong to different families. We randomly select 5% of the folds (approximately 2000 sequences) to form the test set, whereas the remaining data are used for training.

*Signaling protein interaction detection (DPI):* Signaling networks play a crucial role in regulating cellular processes and are often disrupted in diseases such as cancer. Signaling proteins serve as key drug targets, and understanding their interactions is essential for deciphering both healthy and diseased cellular functions. To study these interactions, we utilize the dataset curated by Cunningham *et al.* (2020), which records interactions between signaling proteins and peptides obtained through array-based assays. The training set contains 447 domains and 56 194 peptides, with more than 2 millions interactions recorded. For testing, we focus on signaling interactions involving phosphotyrosines and aim to predict interactions mediated by phosphotyrosine binding (PTB) domains, tyrosine phosphatases (PTP), and tyrosine kinases (TK). Across these 3 groups, we have 58 binding domains and 19 589 peptides.

## 3 Methodology

### 3.1 Discriminative capabilities of pooled representations

In this section, we describe the initial observations and intuition that led us to develop a local pooling strategy for PLM applications. An effective pooling technique should ideally reduce the dimensionality of the data while preserving critical information learned by PLMs. In particular, pooled representations should capture similarities between biological sequences with the same annotations and distinguish between those with different ones. Here, we analyze the representations themselves, independent of further training downstream.

We first compare the CLS-Pooling and Avg-Pooling protein representations from different structural folds of the SCOPe dataset (Fox *et al.* 2014). Proteins in different folds are dissimilar at the sequence and structural levels, and thus should have distinct representations, especially if the pooled representations are to be utilized for homology detection. Figure 1B shows the pairwise cosine similarities among pooled representations of 20 random protein sequences sampled from different folds. While Avg-Pooling representations exhibits a range of cosine similarities between structurally dissimilar proteins, Cls-Pooling representations are extremely close in terms of cosine similarity (above 0.9). This suggests that the embeddings of [Cls] tokens are not ideal for fine-tuning for homolog detection; indeed we observe this in practice, as we describe in Section 4.

We next consider the pooling representations of highly similar proteins that can nevertheless have very different functional activities. In particular, we analyze the pooled representations of 20 green fluorescent protein (GFP) mutants from the **FLUO** dataset (Sarkisyan *et al.* 2016), 10 each with the highest and lowest activity levels. Ideally, these representations should induce a cosine similarity heatmap in which intra-group pairs (diagonal blocks) have significantly higher similarities than inter-group pairs (off-diagonal blocks). However, producing this heatmap pattern is challenging because despite the differences in fluorescence levels across the two groups, these sequences only differ by a few point mutations (Sarkisyan *et al.* 2016). Both Cls-Pooling and Avg-Pooling, which lack sensitivity to minor edits in the sequence, indeed show limited ability to distinguish sequences from different groups, as they do not produce the desired block-diagonal pattern (see Fig. 1C).

We expect that a local pooling method would yield better representations for distinguishing between GFP variants with different fluorescence levels. To confirm this intuition, we consider all possible *k*-mers in these sequences and apply Avg-Pooling on each *k*-mer to obtain a set of pooled representations for each sequence. Next, to compare the representations of two sequences, for each *k*-mer representation in one sequence, we find the *k*-mer representation in the other sequence with the highest cosine similarity to it. This serves as an approximation of the alignment of these *k*-mers in the embedding space. We then identify the minimum distance among these pairs to evaluate the least similar match in the alignment, thus highlighting subtle differences that might not be captured by global pooling methods. Figure 1C (right) further shows the heat map of these pairwise measures (obtained with k=7), in which the 10×10 diagonal block (bottom-left corner) that corresponds to highly active mutants has notably higher similarities than the remaining pairs. Similarities among low fluorescent mutants, however, are not necessarily higher than those of high-low pairs. This is most likely because mutations that abrogate activity are much more variable than those that enhance it. Nonetheless, this result clearly shows a stronger discriminative ability of the local method compared to Avg-Pooling and Cls-Pooling, and thus motivates the use of local pooling methods for PLM applications, which is the major focus of this paper.

### 3.2 Attention pooling

The attention mechanism (see Section 2) can be considered a form of local pooling as it focuses on aggregating features based on localized relevance or importance within a defined scope, rather than treating all elements uniformly across the entire input. The attention-based features can dynamically assign greater importance to specific pairs of residues, which will be particularly beneficial in situations where certain interactions are more critical for the protein’s biological activity than others. This allows the model to extract useful features that focus on these influential pairs, enhancing the downstream model’s ability to learn and represent the underlying biological significance of the sequences. In this section, we propose the application of this mechanism in the context of fine-tuning PLM representations. Depending on the downstream task, this attention pooling mechanism can take different formulations, as described below.

*Self-attention pooling:* In standard supervised learning settings (i.e. regression and classification tasks), where the predictive input is a single protein sequence, we can directly apply the self-attention mechanism mentioned in Section 2, followed by averaging the attention-pooled features. Specifically, letting $H\in R^{l\times d}$ denote the PLM sequence embedding for this input, we define the self-attention pooled representation of H as:


(3) $$P_{SA}(H)≜{\bar{1}}_{l}\times \text{softmax}\left(\frac{Q\left(H\right){\left[K\left(H\right)\right]}^{⊤}}{\sqrt{d}}\right)V\left(H\right)\;,$$

where Q(·), K(·), V(·) are learnable nonlinear transformations (see description in Section 4) and ${\bar{1}}_{l}$ denotes a *l*-dimensional vector filled with 1/l entries, which we use to compute the row average of the attention output (i.e. each row corresponds to one pooled representation). Intuitively, this attention-pooled representation is more versatile than the global Avg-Pooling representation. For example, it could approximate the Avg-Pooling representation by learning the Q(·),K(·),V(·) functions such that large attention weights are assigned to the diagonal terms of the weight matrix (implying that protein function is driven by individual motifs). On the other hand, the learned attention weights could focus on off-diagonal terms, excelling in cases where long-range motif interactions drive protein function.

*Cross-attention pooling:* The attention mechanism can be useful in scenarios where we need to contrast two protein samples, or predict their interactions. In these cases, we can adapt the attention-pooling layer to extract joint features from two distinct sets of pooled embeddings, similar to performing a local alignment in the embedding space. Unlike the self-attention variant that aims to capture intra-sequence dependencies, the cross-attention formulation focuses on the interactions between different localities of either sequence. This ability to cross-reference and align features from two protein samples will facilitate the discovery of structural similarities and differences in motifs, binding sites, or functional domains of the two protein interfaces. Consequently, it will also enable more nuanced predictions of protein-protein interactions, evolutionary relationships, and functional annotations. Letting $H\in R^{l\times d}$ and $H^{\prime }\in R^{l^{\prime }\times d}$ denote a pair of sequence embeddings, their cross-attention pooled representation is given by:


(4) $$P_{CA}(H|H^{\prime })≜{\bar{1}}_{l}\times \text{softmax}\left(\frac{Q\left(H\right){\left[K\left(H^{\prime }\right)\right]}^{⊤}}{\sqrt{d}}\right)V\left(H^{\prime }\right)\;.$$

Due to the asymmetric application of the Q(·),K(·),V(·) transformations on H and $H^{\prime }$, $P_{CA}(H|H^{\prime })$ and $P_{CA}(H^{\prime }|H)$ will be different for every pair of input sequences. As such, in pairwise prediction tasks, the downstream fine-tuning network could be modeled as a comparator function Δ that outputs an interaction/relationship metric given these representations. For example, in the **RH** and **DPI** tasks described in Section 2.3, we could set Δ to be the cosine distance function to contrast pooled embeddings (see Section 3.5).

### 3.3 BoM pooling

A practical caveat of the above attention pooling methods is the quadratic cost in sequence length of computing the self-attention weight matrix $Q(H){\left[K(H)\right]}^{⊤}$, or the cross-attention weight matrix $Q(H){\left[K(H^{\prime })\right]}^{⊤}$. To rectify this problem, our key algorithmic contribution is a novel hierarchical method called bag-of-mer pooling (BoM-Pooling). The BoM-Pooling layer first cascades the input embedding H as a tensor of sliding *k*-mers with stride *s*, similar to common practices in biological modeling. The $i^{\text{th}}$  *k*-mer, which contains all embedding tokens between index s(i−1)+1 and s(i−1)+k inclusive, is denoted by $S_{i}={\left[h_{s(i-1)+j}\right]}_{j=1,\ldots ,k}$. For each *k*-mer, we apply Avg-Pooling to obtain a locally pooled representation $\omega_{i}≜\frac{1}{k}\sum_{j=1}^{k}h_{s(i-1)+j}$. Let n(l,s,k)≜⌊(l−k+1)/s⌋+1 further denote the number of all overlapping *k*-mers in H. Then this intermediate local Avg-Pooling representation is given by:


(5) $$P_{\mathrm{LAvg}}(H;k,s)\;≜[\omega_{1},\omega_{2},\ldots ,\omega_{n(l,s,k)}]\;.$$

The size of this representation depends on the parameters *k* and *s* and also varies from sequence to sequence. To ultimately generate a fixed-size embedding for downstream fine-tuning models, BoM-Pooling further applies attention pooling to this intermediate representation, which now only scales quadratically in the number of *k*-mers. With a favorable trade-off, this can significantly reduces the runtime of attention pooling while maintaining high downstream performance (as shown in Section 4). Specifically, the self-attention and cross-attention BoM-Pooling representations with window size *k* and stride *s* are given by:


(6) $$\begin{array}{c} P_{\mathrm{SBoM}}(H)≜P_{SA}(P_{\mathrm{LAvg}}(H;k,s))\;,\\ P_{\mathrm{CBoM}}(H|H^{\prime })≜P_{CA}(P_{\mathrm{LAvg}}(H;k,s)|P_{\mathrm{LAvg}}(H^{\prime };k,s)). \end{array}$$

An overview of our BoM-Pooling method is given in Fig. 2.

### 3.4 Fidelity-compactness trade-off of BoM-Pooling

Reducing the number of pooled tokens in the intermediate representation intuitively involves a trade-off between preserving the fidelity of the original embedding tokens and improving computational efficiency. Since the unfiltered information in the raw embeddings is critical for downstream fine-tuning (as demonstrated in Section 4, where full attention pooling significantly outperforms other baselines), the success of BoM-Pooling lies in minimizing the dimensionality while maintaining faithfulness to the original tokens. To study this insight, we propose a metric for pooling fidelity, and show that varying the window parameters *k* and *s* indeed simulates a trade-off between pooled representation compactness and fidelity to the original embeddings. The favorability of this trade-off interestingly depends on the variance of the original embedding within each overlapping *k*-mer, which reveals important insights about choosing optimal window settings for each application. We defer this theoretical discussion to Supplementary Appendix S1, and empirically show that choosing the right window size has a positive effect on downstream predictive performance (see Section 4).

### 3.5 Downstream network architectures

Similar to previous pooling methods (see general workflow in Fig. 1A), BoM-Pooling representations will be passed to a downstream, trainable neural network to address specific benchmark tasks (see Section 2.3). Depending on the task group, we use different downstream network architectures. For sequence-wise prediction tasks that aim to infer a real-valued target per protein (e.g. **FLUO** and **BLAC**), we adopt a 3-layer feed-forward neural network with hidden dimensions $d_{\text{pool}}\to 256\to 64\to 1$, where $d_{\text{pool}}$ is the dimension of the pooled representations (see Supplementary Appendix S2). In between each layer, we apply batch normalization and a dropout rate of 0.3. When the pooling layer is learnable (e.g. BoM-Pooling), its parameters will be jointly optimized along with the parameters of the downstream network via minimizing the mean-squared-error loss.

For pairwise prediction tasks that aim to infer interactions or relationships among pairs of proteins (e.g. **RH** and **DPI**), we adopt the contrastive learning framework (Schroff *et al.* 2015) from computer vision to learn representations that can distinguish similar and dissimilar pairs of data. In particular, we sample triplets $\left(H^{a},H^{p},H^{n}\right)$ from the training set, in which the positive sample $H^{p}$ is positively related to the anchor $H^{a}$, whereas the negative sample $H^{n}$ is negatively related to the anchor. For example, a positive label implies a pair of homologs in the **RH** task, or an interacting domain-peptide pair in the **DPI** task. For each sampled triplet, we minimize the contrastive loss function $\text{max}\left(0,\Delta (P(H^{a}),P(H^{p}))-\Delta (P(H^{a}),P(H^{n}))+m\right)$, where Δ is the cosine distance function, P is an arbitrary pooling operator, and *m* is a margin parameter.

For cross-attention BoM-Pooling, the pooled representation is pair-dependent in order to extract cross-sequence attention features (e.g. how amino acids in a binding domain interact with amino acids in the peptide). In other pooling mechanisms, this information would be learned at the sequence level (i.e. after pooling), which would be more challenging since the amino acid tokens would already be collapsed into the pooled representation. In particular, we respectively replace the distance terms $\Delta (P(H^{a}),P(H^{p}))$ and $\Delta (P(H^{a}),P(H^{n}))$ with $\Delta (P_{\mathrm{CBoM}}(H^{a}|H^{p}),P_{\mathrm{CBoM}}(H^{p}|H^{a}))$ and $\Delta (P_{\mathrm{CBoM}}(H^{a}|H^{n}),P_{\mathrm{CBoM}}(H^{n}|H^{a}))$. This loss function aims to make the anchor embedding closer to the positive sample (with respect to the distance metric Δ) than the negative sample by at least some margin *m* (e.g. m=0.6 in all of our contrastive learning experiments). The learned embeddings and distance function can be used to predict homology relationships between unseen pairs of proteins (e.g. we can estimate the likelihood of two test proteins being homologous by taking the negative of their embedding distance). The choice of distance function is agnostic to whether the input pairs are from similar (e.g. **RH**) or different (e.g. **DPI**) classes of sequences, as the embeddings will be trained to align with this criterion. Although alternate distance functions are also possible, and these may yield performance improvements, we do not experiment with this further in this manuscript.

## 4 Results

We compare our proposed BoM-Pooling method to Eos-Pooling, Cls-Pooling, Avg-Pooling, and full attention pooling (i.e. BoM-Pooling with k=1,s=1) when making predictions for all protein analysis applications described in Section 2.3, which are highly diverse from both the biological and machine learning application points of view. To make fair comparisons, where all pooling methods have the same number of trainable parameters, we augment Eos-Pooling, Cls-Pooling, Avg-Pooling with trainable transformations Q(·),K(·),V(·) (using the same number of transformation as used for BoM-Pooling). The trainable pooled representation is then modeled as the sum of the Q(·),K(·),V(·) transformations of the original pooled vector. Since Cls, Avg and Eos pooling are most frequently utilized without these additional trainable parameters, results with these original “vanilla” versions are included in Supplementary Appendix S3.

To demonstrate the robustness of BoM-Pooling, we perform our experiments on 5 different PLMs: ProtBERT (Elnaggar *et al.* 2021), ProtT5-XL (the version trained on UniRef 50) (Elnaggar *et al.* 2021), and ESM-2 models with 35M, 150M, and 650M parameters (Rives *et al.* 2021). Details of these PLMs and all hyper-parameter choices for each task, including window size *k* and stride *s* of the BoM-Pooling method, are reported in Supplementary Appendix S2.

### 4.1 Protein property prediction

In this section, we study the sequence-wise prediction tasks **FLUO** and **BLAC** using embeddings extracted from various pre-trained PLMs. Both tasks can be formulated as supervised regression problems, and are trained using the methodology described in Section 3.5. For each task, we record the average Spearman’s correlation (ρ) across 5 different random seeds.

**Figure 3.**
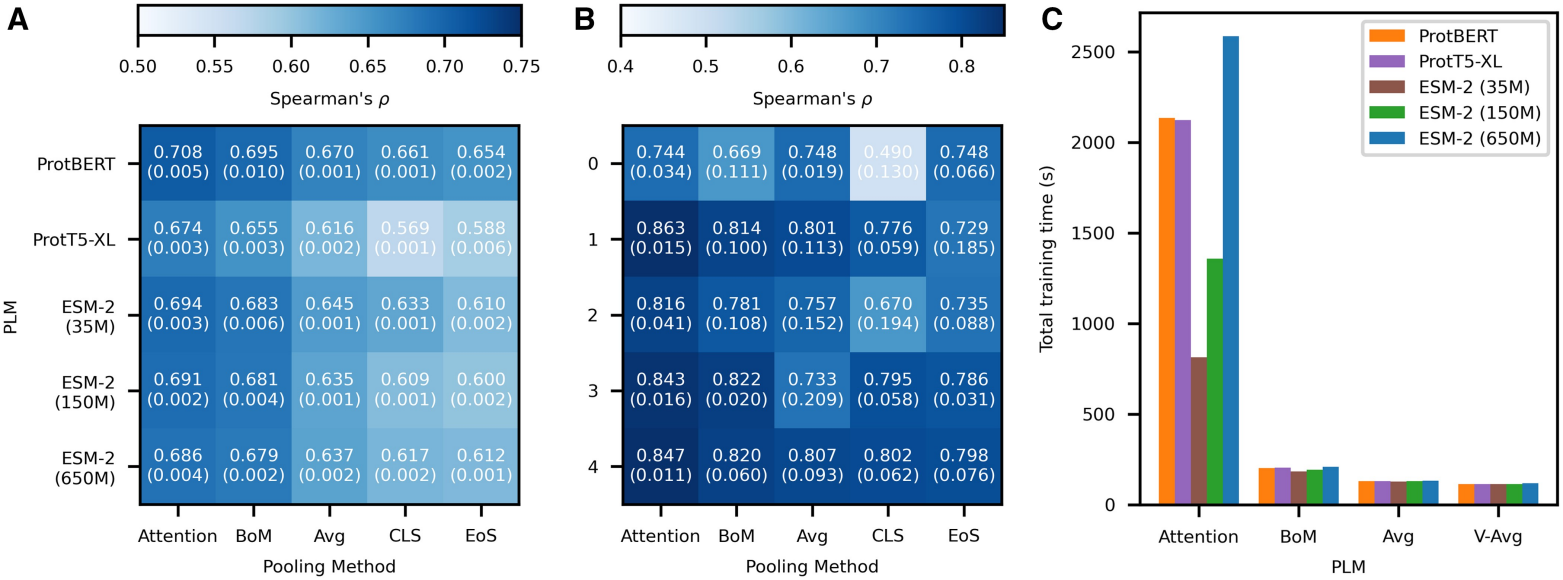
Heatmaps of mean Spearman’s correlation (ρ) over five random seeds using various combinations of pooling techniques and PLMs for (A) Fluorescence intensity prediction and (B) β-lactamase activity level prediction. Each box gives the mean ρ with the number in parentheses indicating the standard deviation. (C) Total training time for **BLAC**. Training time for **FLUO** is given in Supplementary Appendix S3.

For the **FLUO** and **BLAC** tasks, we find that for each of the 5 PLMs, as expected, full attention pooling performs better than all other pooling approaches (Fig. 3A and B). BoM-Pooling is the second best performing method, outperforming all other pooling methods except in one case; further, its performance is remarkably close to that of full attention pooling for **FLUO** (differences in ρ of <0.012 on average over 5 models), with somewhat larger differences for **BLAC** (<0.041 on average over 5 models). Across models, the median increases in correlation coefficients of BoM-Pooling as compared to the best learnable pooling technique (Avg-Pooling for both **BLAC** and **FLUO**) are 1.62% and 6.33%, respectively. Compared to the worst performing learnable pooling technique (EoS-Pooling), these gains are up to 4.58% and 11.4% for **BLAC** and **FLUO**. Further, if we consider the increase of BoM-Pooling as compared to the standard Avg-Pooling and Cls-pooling typically used (i.e. without additional training parameters), we see median performance improvements of 5.89% and 11.3% for **FLUO**. These numbers are 35.5% and 50.7% for **BLAC**. We note that the performance gap between attention and nonattention pooling methods is relatively smaller for ProtBERT than for the other PLMs (and is sometimes negative); this could be due to the fact that ProtBERT is the only PLM we test that uses absolute positional encoding (compared to relative positional encoding in other models). While both full attention pooling and BoM-Pooling have excellent performance, training using full attention pooling is ∼5-12x slower than BoM-Pooling on both the **BLAC** (Fig. 3C) and **FLUO** datasets (Supplementary Fig. 6C), demonstrating that BoM-Pooling has an excellent time versus performance trade-off as compared to full attention pooling.

We next compare our full attention and BoM-Pooling approaches to recent state-of-the art models for these tasks that were published with the DeepProtein benchmark suite (Xie *et al.* 2024) (Table 1). For both tasks, most full attention and BoM-pooling variants outperform the best model from the DeepProtein suite (i.e. CNN, compare numbers in Fig. 3 with Table 1). In contrast, all fine-tuning baselines without attention pooling (i.e. CLS-Pooling, EoS-Pooling and BoM-Pooling) failed to beat the CNN baseline in the FLUO task, clearly demonstrating the benefit of attention pooling in downstream modeling.

**Table 1.** Comparing the the best/worst Spearman’s correlations achieved by two attention-based pooling variants (with five different PLMs) in each task against baseline models made available in the DeepProtein benchmark suite (Xie *et al.* 2024).^a^

Model	FLUO (ρ ↑)	**BLAC** (ρ↑)
CNN	0.680 ± 0.001	0.721±0.020
CNN-RNN	0.678 ± 0.001	0.695±0.012
Transformer	0.648 ± 0.001	0.348±0.002
GCN	0.397 ± 0.002	0.417±0.001
GAT	0.251 ± 0.002	0.196±0.013
NeuralFP	0.413 ± 0.011	0.133±0.020
AttentiveFP	0.260 ± 0.003	0.058±0.011
MPNN	0.237 ± 0.001	0.068±0.015
PAGTN	0.188 ± 0.036	0.092±0.018
Graphormer	0.067 ± 0.002	0.103±0.018
Attention (Best)	0.708 ± 0.005	0.863±0.015
Attention (Worst)	0.674 ± 0.003	0.744±0.034
BoM (Best)	0.695 ± 0.010	0.822±0.020
BoM (Worst)	0.655 ± 0.005	0.669±0.111

a Each result is averaged over five random seeds and the standard deviation is provided. For each task, the highest performance is highlighted in **bold**. The best/worst results from each attention-based pooling variant do not necessarily come from the same PLM base.

### 4.2 Remote homology detection

We trained a contrastive model for each pooling baseline using the methodology described in Section 3.5. To assess remote homology detection performance, we first calculate the likelihood of each test sample being a homolog of any other sample within the test set. Given this list of likelihoods, we can compute the classification AUROC per test sample, with positive labels given to only remote homolog pairs, and negative labels given to nonhomolog pairs. Nonremote homolog pairs are excluded from this evaluation to align with the objective of remote homolog detection; we defer the evaluation that includes these pairs to Supplementary Appendix S4.

**Figure 4.**
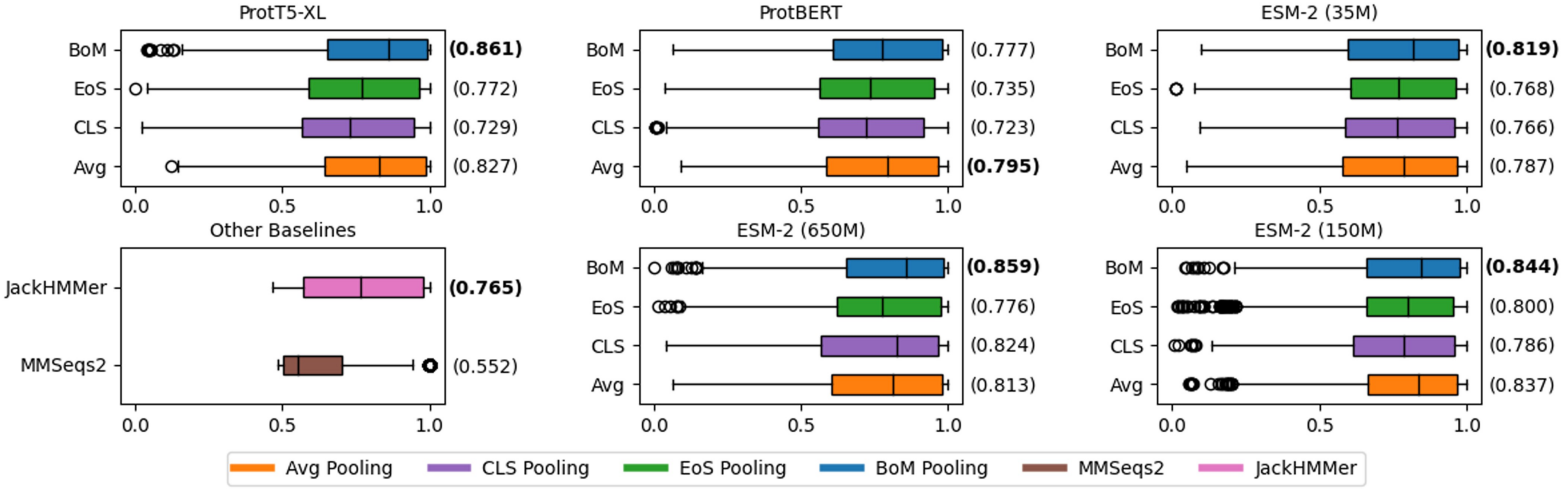
Box plots showing the AUROC for **RH** for each sequence across different combinations of PLMs and pooling methods, as well as two other remote homology detection baselines, MMSeqs2 and JackHMMer. Only remote homolog pairs and nonhomolog pairs are considered in the evaluation. Median AUROC is annotated next to each box, and is highlighted in **bold** if its the best performing pooling method within its respective group.

Figure 4 shows per-sequence AUROC performance for each combination of pooling method and PLM. We omit further characterization of full attention pooling as with appropriate choices of window size, BoM-Pooling well approximates it. We observe that BoM-Pooling with k=100,s=80 outperforms all other pooling baselines except in one case. For example, using the ProtT5-XL model, BoM-Pooling has better median AUROC than the next best method (trainable Avg-Pooling) by 4.11%. We additionally compare our method against two classical baselines, MMSeqs2 (Steinegger and Söding 2017) and JackHMMer (Johnson *et al.* 2010). All BoM-Pooling variants generally outperform both MMSeqs2 and JackHMMer, while having much faster inference time once trained. We note that there is a recent PLM-based approach TM-Vec (Hamamsy *et al.* 2024); we cannot fairly compare our approach to TM-Vec as it is trained on SwissProt and CATH, the latter of which is a structural database that includes homolog pairs that are in our SCOP-derived test set.

### 4.3 Signaling protein interaction detection

Our last experiment studies the effect of BoM-Pooling on predicting signaling protein interactions using contrastive learning. Following the methodology described in Section 3.5, we utilize contrastive learning models for all combinations of the two PLMs that performed best overall in previous experiments [ProtT5-XL and ESM-2 (650M)] and various pooling strategies (Avg-Pooling and BoM-Pooling with k∈{20,40,60,80,100}). We compare these models to 2 non-PLM methods for interaction detection, NetPhorest (Miller *et al.* 2008) and the conventional approach of using position-specific scoring matrices (PSSM) (Stormo *et al.* 1982). The difference in pooling methods only applies to embedding the binding domains, as phosphopeptides are much shorter than the window size *k* of BoM-Pooling. A triplet is composed of one domain as the anchor sample, and two peptides as the positive and negative samples. The contrastive learning training methodology is otherwise similar to that of the remote homology task. All models are trained for 2000 epochs where each will cycle through all domains in the training set.

**Figure 5.**
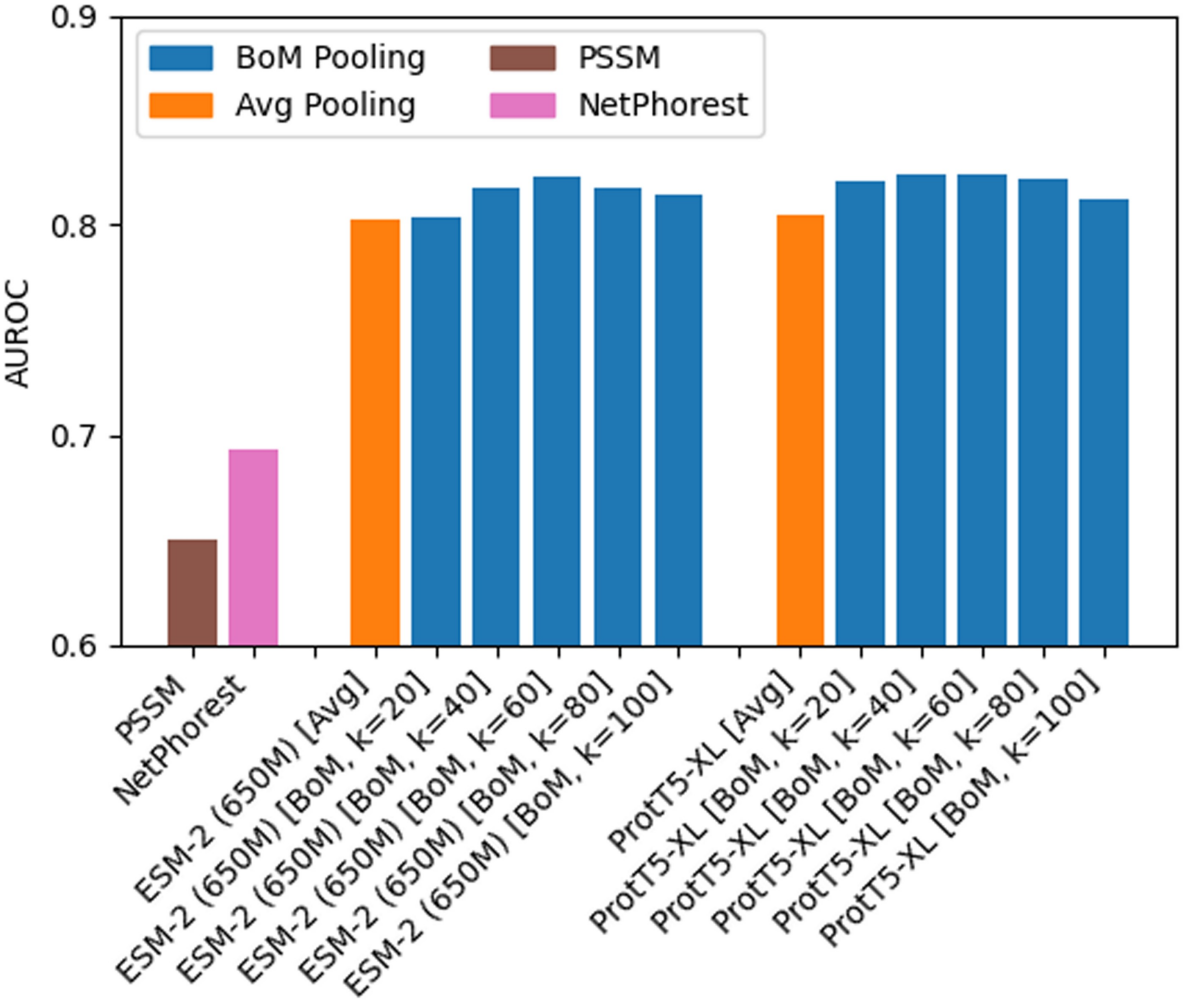
AUROC scores of signaling protein interaction prediction for two baseline methods, PSSM and NetPhorest, and training of embeddings from ESM-2 (650M) and ProtT5-XL using Avg-Pooling and BoM-Pooling as we vary *k* from 20 to 100.

Figure 5 shows the AUROC scores of all approaches listed above on the test set comprised of approximately 130000 phosphotyrosine peptide interactions involving Kinase-TK, PTP, or PTB domains. As anticipated, all fine-tuned PLMs substantially outperform both NetPhorest and PSSM, with up to 26% increase in AUROC. Additionally, we find that BoM-Pooling also consistently outperforms Avg-Pooling across both PLMs, which agrees with the findings from earlier experiments. We additionally observe that the performance of BoM-Pooling peaks at k=60 for both PLMs. While the Kinase-TK, PTP, or PTB domains vary in length from 100–300 AAs, the better performance at k=60 may arise if residues that affect binding are clustered within shorter subsequences of this length. Outside of this application, this observation further suggests that performance can be improved simply by tuning the window setting.

## 5 Conclusions

This paper studies the impact of pooling methods on performance when leveraging PLMs to predict protein properties. Here we propose a new pooling algorithm, BoM-Pooling, a locality-aware hierarchical approach that is especially suitable for protein sequences as it can capture essential local interactions among amino acids in overlapping windows (*k*-mers). We compare BoM-Pooling to previous pooling strategies in 17 different experiments (across 4 different prediction tasks, utilizing up to 5 different PLMs). Our method outperforms all other considered pooling strategies in 15 of them while incurring negligible added runtime over simple Avg-Pooling. This agrees with our theoretical findings (see Supplementary Appendix S1) regarding the “fidelity” of these pooling strategies (i.e. how well these pooling strategies represent the original amino acid embeddings). Thus far, we have utilized BoM-Pooling in the common application where PLM layers are frozen, and the embeddings are utilized in trainable, downstream architectures. We note that our implementation of BoM-Pooling cannot be used to replace inner pooling layers of a PLM in a deep fine-tuning scheme because BoM-Pooling reduces the output dimension of the layer it is applied to. A possible extension worth exploring is to apply BoM-Pooling to only the key and value matrices in the attention mechanism, thus sparsifying computation without changing the output dimension. Other promising future directions include designing additional general-purpose, high-fidelity pooling techniques that are nevertheless motivated by specific properties of biological domains, thus enriching the intersection of computational biology and machine learning.

## Author contributions

Minh Hoang (Conceptualization, Methodology, Software, Writing — original draft), Mona Singh (Conceptualization, Funding Acquisition, Resources, Supervision, Writing — review & editing)

## Supplementary Material

btaf178_Supplementary_Data

## Data Availability

The data underlying this article are available in the DeepProtein repository (https://github.com/jiaqingxie/DeepProtein/), the SCOPe database (https://scop.berkeley.edu/), and the HSM repository curated by Cunningham et al., 2020 (https://proteinpeptide.io/).
