# Supplementary material for: Locality-aware pooling enhances protein language model performance across varied applications
Source: Bioinformatics. 2025 Jul 15;41(Suppl 1):i217–26. doi: 10.1093/bioinformatics/btaf178 (PMC12261463; doi:10.1093/bioinformatics/btaf178)
Supplement: btaf178_Supplementary_Data [file btaf178_supplementary_data.zip › Hoang.Singh.280.sup.1.pdf]

A. Fidelity-compactness trade-off of the intermediate pooled representation

Intuitively, decreasing the number of pooled tokens in the intermediate representation will trade off some degree of fidelity to the raw embedding in favor of computational efficiency. For example, when we set $k = l$, BoM-Pooling reduces to applying self-attention to the globally average-pooled representation, thereby discarding all pairwise interaction features. Conversely, when both window size k and stride s are set to 1 (i.e., no window pooling), BoM-Pooling reverts to full attention pooling and offers no cost saving. To study this insight, we present the following notion of the fidelity of a pooled representation that quantifies how closely each token in the original embedding $\mathbf{H}$ is represented by the pooled tokens.

Definition 1 (Fidelity of Pooled Representation) Let $\mathbf{H} = [\mathbf{h}_1, \mathbf{h}_2, \ldots, \mathbf{h}_l]$ be a length-l PLM sequence embedding tensor, and $\mathcal{P}(\mathbf{H}) = [\boldsymbol{\omega}_1, \boldsymbol{\omega}_2, \ldots, \boldsymbol{\omega}_n]$ be its pooled representation with n pooled tokens. We define the fidelity when $\mathbf{H}$ is represented by $\mathcal{P}(\mathbf{H})$ as the expected negative distance between a random token sampled uniformly from $\mathbf{H}$ and its closest representation in $\mathcal{P}(\mathbf{H})$. Formally, this quantity is given by:

$$\mathcal{F}(\mathbf{H}, \mathcal{P}(\mathbf{H})) \triangleq -\frac{1}{l}\sum_{t=1}^{l} \min_{i=1,\ldots,n} \|\mathbf{h}_t - \boldsymbol{\omega}_i\|^2 . \quad (7)$$

In the following discussion, we will use this quantity to provide mathematical intuitions that characterize the aforementioned trade-off between computational efficiency and expressiveness for the first step of BoM-Pooling. Trivially, when $\mathcal{P}(\mathbf{H}) = \mathcal{P}_{LAvg}(\mathbf{H}; 1, 1) = \mathbf{H}$, each original token is directly preserved in the pooled representation, resulting in the fidelity score being maximized at 0. This also corresponds to the scenario that BoM-Pooling reverts to full attention pooling. Proposition 1 further demonstrates that, for all other valid choices of k and s, the fidelity score of the local Avg-Pooling representation is minimized when $k = l$, thus reducing it to the global Avg-Pooling representation.

Proposition 1 (Fidelity of Local Avg-Pooling and Global Avg-Pooling) *Let $\mathbf{H}$, P_{Avg}, and P_{LAvg} with parameter k, s be defined as above. Then with all $l \geq k \geq s \geq 1$, the following inequality holds:*

$$\mathcal{F}(\mathbf{H}, \mathcal{P}_{LAvg}(\mathbf{H}; k, s)) \geq \mathcal{F}(\mathbf{H}, \mathcal{P}_{Avg}(\mathbf{H})) . \quad (8)$$

Proof: For simplicity of presentation, we assume that the embedding dimension is $d = 1$. The proof below is extendable to the multivariate case via additivity of variance across dimensions. We also assume that the sliding k-mers perfectly cover the entire sequence, as this can be guaranteed by setting $s \leq k$ and zero-padding to account for the last window. As Avg-Pooling has exactly one pooled representation, the information loss of Avg-Pooling is simply the negative variance of the embedding tokens, that is:

$$\mathcal{F}(\mathbf{H}, \mathcal{P}_{Avg}(\mathbf{H})) \triangleq -\frac{1}{l}\sum_{t=1}^{l}\left\|\mathbf{h}_t - \frac{1}{l}\sum_{i=1}^{l}\mathbf{h}_i\right\|^2 = -\mathrm{Var}[\mathbf{X}] , \quad (9)$$

where $\mathbf{X}$ is a random token drawn uniformly from the set $\{\mathbf{h}_1, \mathbf{h}_2, \ldots, \mathbf{h}_l\}$. On the other hand, we have the following decomposition for the fidelity score of local Avg-Pooling:

$$\begin{aligned} &\mathcal{F}(\mathbf{H}, \mathcal{P}_{LAvg}(\mathbf{H}; k, s)) \\ \triangleq\ & -\frac{1}{l}\sum_{t=1}^{l} \min_{i=1,\ldots,n(l,s,k)} \|\mathbf{h}_t - \boldsymbol{\omega}_i\|^2 \\ =\ & -\frac{1}{l}\sum_{t=1}^{l} \min_{i=1,\ldots,n(l,s,k)} \left\|\mathbf{h}_t - \frac{1}{k}\sum_{j=1}^{k}\mathbf{h}_{s(i-1)+j}\right\|^2 \\ \geq\ & -\frac{1}{l}\sum_{t=1}^{l}\left\|\mathbf{h}_t - \frac{1}{k}\sum_{j=1}^{k}\mathbf{h}_{s\lfloor \frac{t}{s}\rfloor + j}\right\|^2 \\ \geq\ & -\frac{k}{l}\sum_{i=1}^{n(l,s,k)} \underbrace{\frac{1}{k}\sum_{t=1}^{k}\left\|\mathbf{h}_{s(i-1)+t} - \frac{1}{k}\sum_{j=1}^{k}\mathbf{h}_{s(i-1)+j}\right\|^2}_{\mathrm{Var}[\mathbf{X}|\mathbf{X}\in\mathbf{S}_i]} , \end{aligned} \quad (10)$$

where $\mathbf{S}_i$ is the set of all tokens in the i-th k-mer of $\mathbf{H}$. In the above derivation, the first inequality is due to the fact that the minimum across all values of i is less than or equal to setting $i = \lfloor \frac{t}{s} \rfloor$ (i.e.,

Task	window size k	stride s	contrastive margin m	learning rate η (initial/minimum)	batch size b
**FLUO**	100	20	N/A	1e−4/1e−6	48
**BLAC**	100	20	N/A	1e−4/1e−6	48
**RH**	100	80	0.6	1e−4/1e−6	256
**DPI**	$[20, 40, 60, 80, 100]$	$\lceil k/5 \rceil$	0.6	1e−4/1e−6	128

Table 2. Hyper-parameter choices for each task group.

Module	ProtT5-XL (UniRef 50)	ProtBERT	ESM-2 (35M)	ESM-2 (150M)	ESM-2 (650M)	Trainable Pooling Net	Prediction Network
Architecture	PLM	PLM	PLM	PLM	PLM	3-head MLP	MLP
No. params	1.2B	420M	34M	149M	652M	$(1024 + d_{plm}) \times 256$	270K
Output dims	1024	1024	480	640	1280	1024	1

Table 3. Architectures of various networks used in implementation. For ProtT5-XL, we give the number of parameters for the encoder portion of the model only.

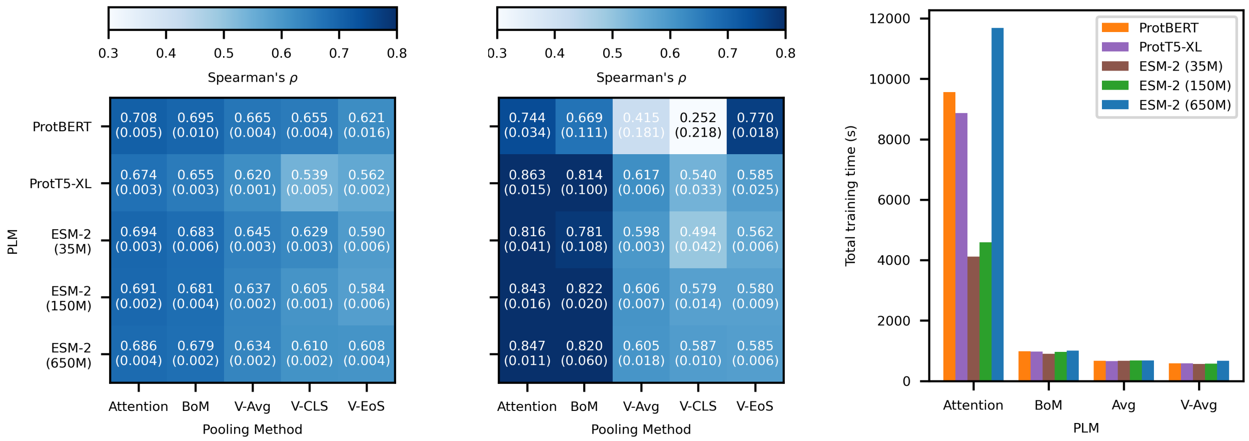

(**A**) Fluorescence (**B**) β-lactamase (**C**) Fluorescence training time

Fig. 6: Heatmaps of mean Spearman's correlation (ρ) over 5 random seeds using various combinations of pooling techniques and PLMs for **(A)** Fluorescence intensity prediction and **(B)** β-lactamase activity level prediction. Each box gives the mean ρ with the number in parentheses indicating the standard deviation. **(C)** Total training time for **FLUO**.

choosing a window that overlaps $\mathbf{h}_t$). This results in a summation over l distances, each of which is between one token embedding and the corresponding window mean. The second inequality then lower-bounds this by a nested sum in which each token can appear in more than one distance terms. Finally, by law of total variance, we have the following inequality:

$$
\begin{aligned}
-\mathrm{Var}[\mathbf{X}] \quad &\leq \quad -\mathbb{E}_{\mathcal{S}_i}\left[\mathrm{Var}[\mathbf{X}|\mathbf{X}\in\mathcal{S}_i]\right] - \underbrace{\mathrm{Var}(\mathbb{E}[\mathbf{X}|\mathbf{X}\in\mathcal{S}_i])}_{\geq 0} \\
&\leq \quad -\sum_{i=1}^{n(l,s,k)} \Pr(\mathbf{X}\in\mathcal{S}_i)\mathrm{Var}\left[\mathbf{X}|\mathbf{X}\in\mathcal{S}_i\right] \\
&= \quad -\frac{k}{l}\sum_{i=1}^{n(l,s,k)} \mathrm{Var}\left[\mathbf{X}|\mathbf{X}\in\mathcal{S}_i\right] \ . \qquad (11)
\end{aligned}
$$

Combining Eq. (9), (10), (11) concludes the proof. □

Remark. The gap between the global average pooling fidelity $\mathcal{F}(\mathbf{H}, \mathcal{P}_{Avg}(\mathbf{H}))$ and local average pooling fidelity $\mathcal{F}(\mathbf{H}, \mathcal{P}_{LAvg}(\mathbf{H}; k, s))$ is controlled by the variance of k-mer mean embeddings $\mathrm{Var}(\mathbb{E}[\mathbf{X}|\mathbf{X} \in \mathcal{S}_i])$. As this term grows larger (via the choice of k and s), $\mathcal{P}_{LAvg}(\mathbf{H}; k, s)$ will be more faithful to the original embedding $\mathbf{H}$ than $\mathcal{P}_{Avg}(\mathbf{H})$. This bound thus sheds insights into controlling the fidelity-compactness trade-off via balancing between minimizing k and maximizing the resulting variance term per input sequence, which could be investigated in future works.

B. Implementation details

We implement our method and all baselines using PyTorch. All experiments are deployed on A100 GPUs. All trainable network weights are optimized using the Adam optimizer [21] with an adaptive learning rate scheduled using cosine annealing with warm restarts [24]. Each learnable transformation Q, K, V in the attention-pooling layer (see Section 3.3) is parameterized by a 2-layer perceptron with hidden dimensions $d \to 256 \to 1024$, where d is the embedding dimension of the PLM. This bottleneck architecture is inspired by popular deep models such as ResNet [13], and empirically gives the best general performance across all benchmark tasks. Our implementation is available at https://github.com/Singh-Lab/bom-pooling.

We give the hyper-parameter choices for each task group in Table 2. These hyper-parameters are chosen via running grid search on a smaller validation dataset. Table 3 describes the architectures of various networks used in our implementation. We note that ProtT5-XL uses a T5 encoder/decoder architecture, but only the encoder is used to embed protein sequences. The number of effective parameters for our use case is therefore fewer than 1.2B. The PLM parameters are not updated during downstream fine-tuning. The trainable pooling network scales with the output dimension d_{plm} of the PLM model in use. The prediction head network only applies to **FLUO** and **BLAC** tasks.

C. Other regression results

For completeness, we also compare the performance of BoM-Pooling to the "vanilla", non-trainable variants of other pooling baselines, which we labeled as V-Cls, V-Eos, and V-Avg. Specifically, Fig. 6A,B replicates the heat maps in Fig. 3, replacing trainable pooling variants with these "vanila" baselines. As expected, all of the trainable variants outperformed their "vanila" counterparts, and thus we observe a clear margin between the performance of BoM-Pooling and these methods. Fig. 6C reports the total training time of various baselines on the **FLUO** task, which again confirms the significant speedup of BoM-Pooling.

D. Other remote homology detection results

We first replicate the per-sequence AUROC box plots for the scenario that involves all test pairs (e.g., both remote and non-remote homolog pairs are given positive labels, whereas non-homolog pairs are given negative labels). In this scenario, we observe that both MMSeqs2 and JackHMMer achieved significantly higher median AUROC scores than in Fig. 4. This

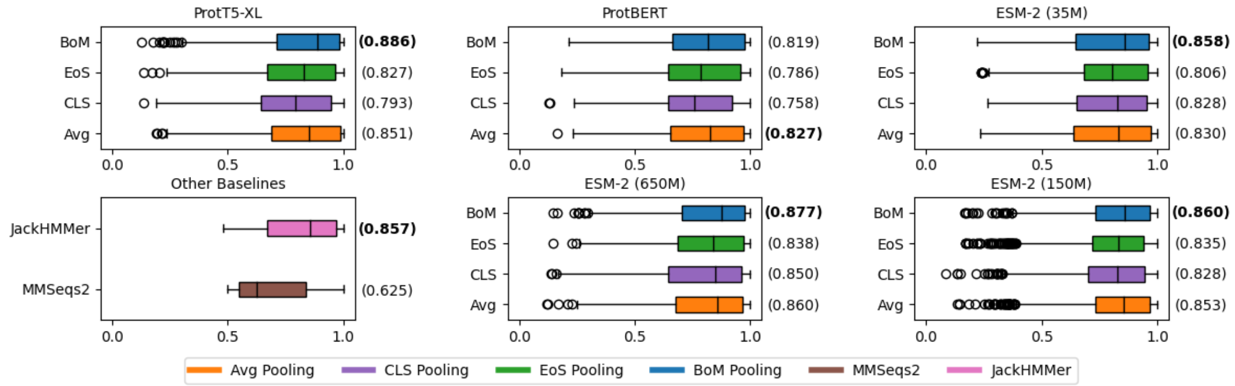

Fig. 7: Box plots showing the AUROC for remote homology classification for each sequence across different combinations of PLMs and pooling methods, as well as two other remote homology detection baselines, MMSeqs2 and JackHMMer. All test pairs are considered in the evaluation. Median AUROC is annotated next to each box, and is highlighted in **bold** if its the best performing pooling method within its respective group.

is most likely because non-remote homolog pairs have relatively higher sequence similarities, and thus are easier to detect by alignment-based approaches. On the other hand, all fine-tuning baselines tend to retain similar performance, indicating their robustness to low sequence similarities.
